# Dnmt3aa but Not Dnmt3ab Is Required for Maintenance of Gametogenesis in Nile Tilapia (*Oreochromis niloticus*)

**DOI:** 10.3390/ijms221810170

**Published:** 2021-09-21

**Authors:** Feilong Wang, Zuliang Qin, Zhiqiang Li, Shuangyi Yang, Tian Gao, Lina Sun, Deshou Wang

**Affiliations:** Key Laboratory of Freshwater Fish Reproduction and Development (Ministry of Education), Key Laboratory of Aquatic Science of Chongqing, School of Life Sciences, Southwest University, Chongqing 400715, China; feilongwang0720@163.com (F.W.); qzuliang1996@163.com (Z.Q.); lzq07010622@163.com (Z.L.); ysy631104725@163.com (S.Y.); 18375621383@163.com (T.G.)

**Keywords:** Nile tilapia, DNA methylation, *Dnmt3a*, gonad development, gametogenesis

## Abstract

*Dnmt3a*, a de novo methyltransferase, is essential for mammalian germ line DNA methylation. Only one *Dnmt3a* is identified in mammals, and homozygous mutants of *Dnmt3a* are lethal, while two *Dnmt3a* paralogs, *dnmt3aa* and *dnmt3ab*, are identified in teleosts due to the third round of genome duplication, and homozygous mutants of *dnmt3aa* and *dnmt3ab* are viable in zebrafish. The expression patterns and roles of *dnmt3aa* and *dnmt3ab* in gonadal development remain poorly understood in teleosts. In this study, we elucidated the precise expression patterns of *dnmt3aa* and *dnmt3ab* in tilapia gonads. *Dnmt3aa* was highly expressed in oogonia, phase I and II oocytes and granulosa cells in ovaries and spermatogonia and spermatocytes in testes, while *dnmt3ab* was mainly expressed in ovarian granulosa cells and testicular spermatocytes. The mutation of *dnmt3aa* and *dnmt3ab* was achieved by CRISPR/Cas9 in tilapia. Lower gonadosomatic index (GSI), increased apoptosis of oocytes and spermatocytes and significantly reduced sperm quality were observed in *dnmt3aa*^−/−^ mutants, while normal gonadal development was observed in *dnmt3ab*^−/−^ mutants. Consistently, the expression of apoptotic genes was significantly increased in *dnmt3aa*^−/−^ mutants. In addition, the 5-methylcytosine (5-mC) level in *dnmt3aa*^−/−^ gonads was decreased significantly, compared with that of *dnmt3ab*^−/−^ and wild type (WT) gonads. Taken together, our results suggest that *dnmt3aa*, not *dnmt3ab*, plays important roles in maintaining gametogenesis in teleosts.

## 1. Introduction

DNA methylation, a mechanism of epigenetics, plays a crucial role in the control of development-related gene expression during gametogenesis and early embryogenesis [1,2]. During germ cell development, epigenetic reprogramming occurs dynamically, remodeling DNA methylation marks in particular [3,4]. At day 7.5 of early embryogenesis (E7.5) in mice (*Mus musculus*), global DNA methylation of primordial germ cells (PGCs) is erased. Later, de novo DNA methylation proceeds differentially between male and female germ cells, earlier in spermatogenesis than in oogenesis. In female germ cells, de novo DNA methylation occurs in arrested oocytes in meiotic prophase I. However, in male germ cells, it takes place in mitotically arrested prespermatogonia before birth [5,6,7].

DNA methylation is catalyzed by a group of enzymes called DNA methyltransferases (*dnmts*), including *Dnmt1* and *Dnmt3*. *Dnmt1* is involved in the methylation of hemimethylated DNA and thus called maintenance DNA methyltransferase, while *Dnmt3* is able to place methylation marks on previously unmethylated CpGs of DNA and thus is mainly responsible for de novo DNA methylation during development [8,9]. In mammals, the *Dnmt3* subfamily is composed of three members, *Dnmt3a*, *Dnmt3b* and *Dnmt3l* [10]. Recently, *Dnmt3c*, a novel rodent-specific member of the de novo *dnmts*, has been identified to regulate DNA methylation in the male germline [11]. Of these, *Dnmt3a*, *Dnmt3b* and *Dnmt3c* have been proven to have catalytic activities in vivo, whereas *Dnmt3l* is a catalytically inactive DNA methyltransferase cofactor [9,12].

Studies on mammals have shown that *Dnmt3a*, *Dnmt3c* and *Dnmt3l*, not *Dnmt3b*, are required for gametogenesis. During the development of male germ cells in mice, *Dnmt3a* exhibited dynamic expression patterns, and it is highly expressed in spermatogonia and spermatocytes [13,14]. Male germ cells without *Dnmt3a* or *Dnmt3l* undergo meiotic failure, impaired spermatogenesis, which results in no spermatocytes, spermatids or spermatozoa, and significantly reduced testis size in *Dnmt3a* conditional mutant mice [15,16,17,18]. In female mice, *Dnmt3a* is expressed in follicles and stromal cells at different developmental stages, and exists in the cytoplasm and nucleus of oocytes and granulosa cells [19,20]. *Dnmt3a* or *Dnmt3l* null oocytes fail to acquire methylation during oocyte growth, which leads to abnormal embryonic development after fertilization [15,17].

Teleost-specific whole genome duplication has increased the *dnmts* copy number [21,22]. Two *Dnmt3a* paralogous genes (*dnmt3aa* and *dnmt3ab*) have been identified in Nile tilapia (*Oreochromis niloticus*), zebrafish (*Danio rerio*), flatfish (*Solea senegalensis*) and ricefield eel (*Monopterus albus*) [21,23,24,25]. Studies on *dnmt3aa* and *dnmt3ab* in fish have been mainly focused on their expression in gonads. In Nile tilapia and bluehead wrasse (*Thalassoma bifasciatum*), the expressions of *dnmt3aa* and *dnmt3ab* are significantly higher in the testes than in the ovaries, and significantly increased during the sex reversal from female to male induced by Fadrozole [26] or social cues [27,28]. In ricefield eel, *dnmt3aa* and *dnmt3ab* are highly expressed in spermatocytes of testes, with the expression of *dnmt3aa* significantly increased during the female to male sex reversal [25,29]. These studies suggest that *dnmt3aa* and *dnmt3ab* may play important roles in the gonadal development of fish; however, their detailed expression profiles during the sex determination and differentiation and gonadal development of teleosts are unclear. Recently, some research groups reported the mutation of *dnmt3aa* and *dnmt3ab* in zebrafish, demonstrating their critical function on behavior regulation [30], temperature adaptation [31] and brain neural development [32]. Nevertheless, the roles of *dnmt3aa* and *dnmt3ab* in the gonadal development of fish remain unknown.

Nile tilapia belongs to Perciformes, Cichlidae. Fast growth speed, strong adaptability and disease resistance make it an important aquaculture fish in the world. It is considered to be an excellent model for studying gene expression and function related to reproduction and fertility due to its short spawning cycle (14 days), stable XX–XY sex determination system, availability of genetic all-XX and all-XY fish [33] and high-quality genome sequences [34]. In particular, genome editing by CRISPR/Cas9 techniques established in our research group has contributed to the research of gene function in Nile tilapia [35]. Generally, several key biological events occur at different time points during gonadal development in Nile tilapia, such as sex determination and differentiation at 5–10 dah (days after hatching), the initiation of germ cell meiosis and oogenesis in the XX gonads (ovaries) at 30 dah, the initiation of spermatogenesis in the XY gonads (testes) at 75–90 dah and sperm maturation in the XY gonads and vitellogenesis in the XX gonads at 180 dah [36]. In the present study, we clarified the precise expression profiles of *dnmt3aa* and *dnmt3ab* during the key stages of gonadal development in females and males and uncovered their roles in reproduction and fertility in teleosts and the possible mechanism involved by homozygous mutant establishment and phenotype analyses in Nile tilapia.

## 2. Results

### 2.1. Expression Patterns and Cellular Localization of Dnmt3aa and Dnmt3ab in Developing Gonads

Ontogeny analyses showed that *dnmt3aa* and *dnmt3ab* displayed sexually dimorphic expression profiles in developing gonads (Supplementary Figure S1). *Dnmt3aa* and *dnmt3ab* were expressed in gonads from as early as 5 dah, with relatively higher expression in the testes than the ovaries. In the testes, *dnmt3aa* was up-regulated from 5 to 180 dah. In the ovaries, *dnmt3aa* was up-regulated from 5 dah and reached the highest level at 120 dah, and maintained this level till 180 dah (Supplementary Figure S1a). *Dnmt3ab* was up-regulated from 5 to 120 dah and maintained at a relatively high level in ovaries and testes at 120 and 180 dah (Supplementary Figure S1b). Generally, higher expression was observed for *dnmt3aa* than *dnmt3ab* at all the time points examined.

**Figure 1:**
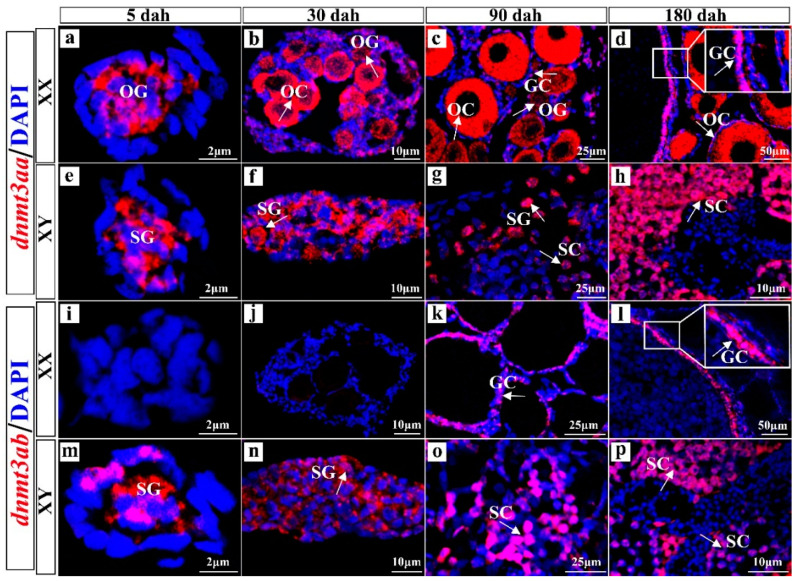
Cellular localization of *dnmt3aa* (**a**–**h**) and *dnmt3ab* (**i**–**p**) in developing gonads by fluorescence in situ hybridization. *Dnmt3aa* was widely expressed in the gonads of Nile tilapia, especially in oogonia, phase I and II oocytes and granulosa cells of ovaries (**a**–**d**) and spermatogonia and spermatocytes of testes (**e**–**h**). *Dnmt3ab* was mainly expressed in granulosa cells of ovaries (**i**–**l**) and spermatocytes of testes (**m**–**p**). OC, oocytes; OG, oogonia; GC, granulosa cells; SC, spermatocytes; SG, spermatogonia; dah, days after hatching. Red fluorescence represents the *dnmt3aa* and *dnmt3ab* signals. Blue fluorescence represents the DAPI signals. Arrows indicate the positive signals. White boxes indicate the regions magnified in (**d**,**i**).

Cell populations expressing *dnmt3aa* and *dnmt3ab* in gonads were identified by fluorescence in situ hybridization (FISH). *Dnmt3aa* was widely expressed in the gonads of Nile tilapia, highly expressed in oogonia, phase I and II oocytes and granulosa cells of ovaries (Figure 1a–d), and spermatogonia and spermatocytes of testes (Figure 1e–h). *Dnmt3ab* was mainly expressed in granulosa cells of ovaries (Figure 1i–l) and spermatocytes of testes (Figure 1m–p). In contrast, no signal for *dnmt3aa* and *dnmt3ab* mRNA was detected in the gonads using the sense probe (Supplementary Figure S2).

### 2.2. Establishment of Nile Tilapia Dnmt3aa and Dnmt3ab Mutant Lines by CRISPR/Cas9

**Figure 2:**
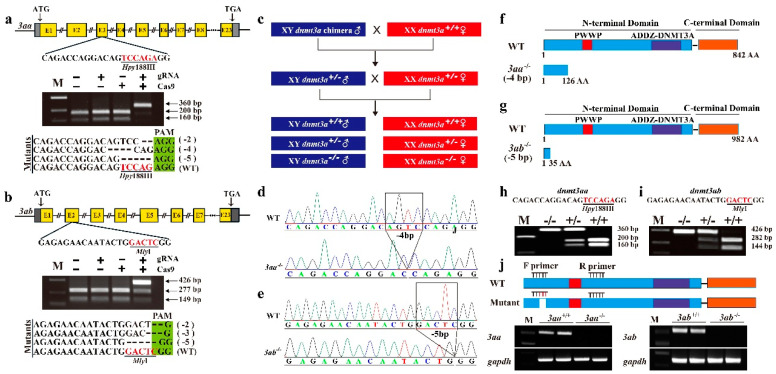
Establishment of *dnmt3aa* and *dnmt3ab* mutant lines. (**a**,**b**) Gene structure of *dnmt3aa* and *dnmt3ab* showing the target site and the *Hpy* 188III and *Mly* I restriction site. The Cas9 mRNA and gRNA were added as indicated. Sanger sequencing results from the uncleaved bands are listed. The PAM is marked in light green. Deletions are marked by dashes (-) and numbers to the right of the sequences in parentheses indicate the loss of bases for each allele. The mutant fish that carried 4 and 5 base-pair deletions were used for homozygous mutant construction of *dnmt3aa* and *dnmt3ab*, respectively. WT, wild type. (**c**) Schematic diagram showing the breeding plans of *dnmt3aa* and *dnmt3ab* F0 to F2 fish. (**d**,**e**) Sequencing results of *dnmt3aa* and *dnmt3ab* genes from WT and homozygous mutant fish. (**f**,**g**) Schematic diagram of Dnmt3aa and Dnmt3ab wild type (WT) and the predicted truncated protein. (**h**,**i**) Identification of F2 genotypes by restriction enzyme digestion assay. (**j**) RT-PCR analysis of *dnmt3aa* and *dnmt3ab* mRNA expression in gonads of mutants and WT fish. The 3′ sequences of forward primer were designed on the target site, which is indicated by white box. No band was amplified in the homozygous mutants, while one band corresponding to *dnmt3aa* and *dnmt3ab* mRNA was amplified in the WT XY testes. *gapdh* was used as internal control.

The guide RNA (gRNA) sites containing *Hpy* 188III and *Mly* I adjacent to protospacer adjacent motif (PAM) were selected in the third and second exon of *dnmt3aa* and *dnmt3ab*, respectively (Figure 2a,b). Complete digestion of the PCR products from *dnmt3aa* and *dnmt3ab* with *Hpy* 188III and *Mly* I, respectively, produced two fragments in the control groups, while an intact DNA fragment was observed in embryos injected with both Cas9 mRNA and target gRNA (Figure 2a,b). Representative Sanger sequencing results from the uncleaved bands were listed. In-frame and frame-shift deletions induced at the target sites were confirmed by Sanger sequencing (Figure 2a,b).

F1 generation fish were obtained by crossing chimeric XY F0 males and WT XX females. Heterozygous F1 offspring with a deletion of 4 bp for *dnmt3aa* and 5 bp for *dnmt3ab* were selected to breed the F2 generation (Figure 2c). Further, homozygous mutant fish of *dnmt3aa* and *dnmt3ab* were validated by Sanger sequencing (Figure 2d,e). Frame-shift mutations led to premature termination of the translation of *dnmt3aa* at amino acid 126 and *dnmt3ab* at amino acid 36 (Figure 2f,g). Restriction enzyme digestion assay identified the *dnmt3aa*^+/+^, *dnmt3aa*^+/−^, *dnmt3aa*^−/−^, *dnmt3ab*^+/+^, *dnmt3ab*^+/−^ and *dnmt3ab*^−/−^ individuals (Figure 2h,i). The loss of *dnmt3aa* and *dnmt3ab* mRNA was confirmed by reverse transcription PCR (RT-PCR) using a specific primer on the target site (Figure 2j).

### 2.3. Gonadal Morphology and Histology of Dnmt3aa^−/−^ and Dnmt3ab^−/−^ Female Mutants

**Figure 3:**
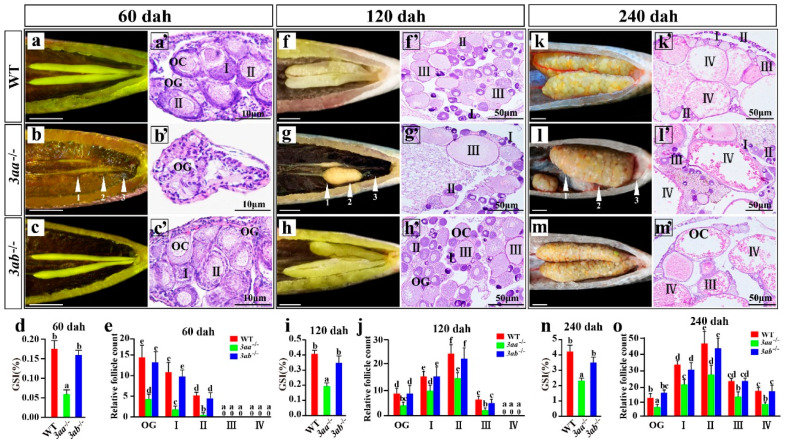
Morphological and histological analyses of WT, *dnmt3aa*^−/−^ and *dnmt3ab*^−/−^ ovaries at 60, 120 and 240 dah. (**a**–**c**,**a**’–**c**’,**f**–**h**,**f**’–**h**’,**k**–**m**,**k**’–**m**’) Morphological and histological observation. (**d**,**i**,**n**) Gonadosomatic index (GSI) (*n* = 10 for each genotype). (**e**,**j**,**o**) Statistical analysis of germ cell counting (*n* = 5 for each genotype). Follicles from the median sections (part2) of ovaries were counted for statistical analyses. (**a**–**c**,**f**–**h**) Gonads were fixed with Bouin’s solution. Different letters above the error bar indicate statistical differences at *p* < 0.05 as determined by one-way ANOVA followed by Tukey’s post hoc test. Results are presented as mean ± SD in (**d**,**e**,**i**,**j**,**n**,**o**). Scale bar in (**a**–**c**,**f**–**h**,**k**–**m**), 1 cm. dah, days after hatching; OC, oocytes; OG, oogonia.

Morphological observation showed that the *dnmt3aa*^−/−^ ovaries atrophied at 60 dah, while there was no difference in gonad morphology between *dnmt3ab*^−/−^ and WT (Figure 3a–c). We selected three different sampling points (part1, part2 and part3) from *dnmt3aa*^−/−^ ovaries, including the smaller gonad part, the thicker part and the atrophy part, for histological observation. The results showed that at 60 dah, WT and *dnmt3ab*^−/−^ ovaries were full of oogonia and phase I and phase II oocytes, while *dnmt3aa*^−/−^ ovaries had only a few oogonia and oocytes (Figure 3a’–c’). Consistently, statistical analysis showed that the GSI of *dnmt3aa*^−/−^ fish was significantly lower than that of WT fish, and the number of follicles at different developmental stages was significantly reduced, while there was no significant difference between *dnmt3ab*^−/−^ and WT fish (Figure 3d,e).

Two more developmental stages at 120 dah and 240 dah were further analyzed. Morphological observations showed that *dnmt3aa*^−/−^ ovaries were still atrophied and degenerated, while *dnmt3ab*^−/−^ ovaries developed normally (Figure 3f–h,k–m). Histological observation showed that the WT and *dnmt3ab*^−/−^ ovaries were filled with phase II and phase III oocytes at 120 dah, while only a few phase II and phase III oocytes existed in *dnmt3aa*^−/−^ ovaries (Figure 3f’–h’). At 240 dah, the oocytes of WT, *dnmt3aa*^−/−^ and *dnmt3ab*^−/−^ females were matured, and less oogonia and phase I, phase II, phase III and phase IV follicle cells were observed in *dnmt3aa*^−/−^ ovaries than the *dnmt3ab*^−/−^ and WT ovaries (Figure 3k’–m’). Consistently, statistical analysis showed that the GSI and the number of follicles of *dnmt3aa*^−/−^ were significantly decreased (Figure 3i,j,n,o). In addition, the number of follicles in the *dnmt3aa*^−/−^ ovarian smaller part (part1) and atrophy part (part3) was also significantly reduced (Supplementary Figure S3). Taken together, these results suggest that the homozygous mutation of *dnmt3aa* resulted in reduced follicles and ovarian atrophy and degeneration in Nile tilapia.

### 2.4. Gonadal Morphology and Histology of Dnmt3aa^−/−^ and Dnmt3ab^−/−^ Male Mutants

**Figure 4:**
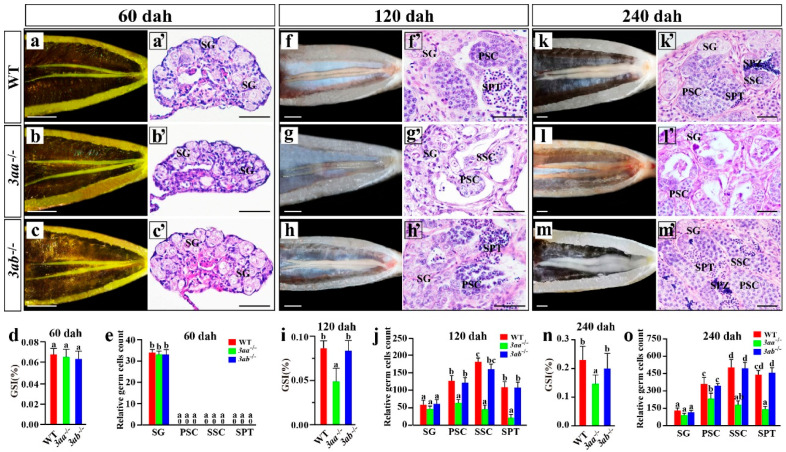
Morphological and histological analyses of WT, *dnmt3aa*^−/−^ and *dnmt3ab*^−/−^ testes at 60, 120 and 240 dah. (**a**–**c**,**a***’*–**c***’*,**f**–**h**,**f***’*–**h***’*,**k**–**m**,**k***’*–**m***’*) Morphological and histological observation. (**d**,**i**,**n**) Gonadosomatic index (GSI) (*n* = 10 for each genotype). (**e**,**j**,**o**) Statistical analysis of germ cell counting (*n* = 5 for each genotype). Germ cells from the median sections of testes were counted for statistical analyses. (**a**–**c**) Gonads were fixed with Bouin’s solution. Different letters above the error bar indicate statistical differences at *p* < 0.05 as determined by one-way ANOVA followed by Tukey’s post hoc test. Results are presented as mean ± SD in (**d**,**e**,**i**,**j**,**n**,**o**). Scale bar in (**a**–**c**,**f**–**h**,**k**–**m**), 1 cm. Scale bar in (**a**’–**c**’,**f**’–**h**’,**k**’–**m**’), 10 μm. dah, days after hatching; PSC, primary spermatocytes; SG, spermatogonia; SPT, spermatids; SPZ, spermatozoa; SSC, secondary spermatocytes.

At 60 dah, no obvious difference was observed in the testicular morphology among them (Figure 4a–c). Histological examination showed that the testes of WT, *dnmt3aa*^−/−^ and *dnmt3ab*^−/−^ fish were full of spermatogonia (Figure 4a’–c’) and with no difference in number (Figure 4e). At 120 dah, the testes of *dnmt3aa*^−/−^ fish were smaller and more transparent than those of the WT fish (Figure 4f–h). Histological examination showed that the testes of WT and *dnmt3ab*^−/−^ fish were full of spermatogenic cells at different developmental stages, while less spermatocytes were observed in *dnmt3aa*^−/−^ testes (Figure 4f’–h’). Statistical analysis showed that the GSI and the number of spermatocytes in the testes of *dnmt3aa*^−/−^ fish were significantly lower than those of WT fish (Figure 4i,j). At 240 dah, morphological observation showed that the testes of the *dnmt3aa*^−/−^ fish, but not the WT and *dnmt3ab*^−/−^ fish, were transparent (Figure 4k–m). Histological examination showed that *dnmt3aa*^−/−^ fish had fewer spermatocytes (Figure 4l’) compared with the *dnmt3ab*^−/−^ fish and the WT fish (Figure 4m’). Consistently, compared with the WT fish, the GSI and spermatocyte number of *dnmt3aa*^−/−^ fish were significantly reduced at 240 dah (Figure 4n,o).

### 2.5. Germ Cell Apoptosis in Dnmt3aa^−/−^ and Dnmt3ab^−/−^ Gonads

**Figure 5:**
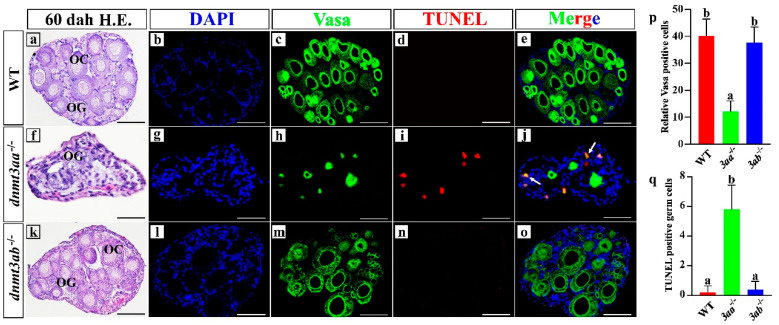
Apoptosis detection in WT, *dnmt3aa*^−/−^ and *dnmt3ab*^−/−^ ovaries at 60 dah. Increased apoptosis of germ cells in the ovaries of *dnmt3aa*^−/−^ fish at 60 dah. (**a**,**f**,**k**) Histological analyses of germ cells by H&E staining. (**b**,**g**,**l**) Nuclei were counterstained with DAPI. (**c**,**h**,**m**) Green fluorescence represents the Vasa signals. (**d**,**i**,**n**) Red fluorescence represents the TUNEL-positive signals. (**e**,**j**,**o**) Co-localization of some Vasa and TUNEL signals as indicated by orange color in Nile tilapia ovaries. (**p**) Germ cell count of WT, *dnmt3aa*^−/−^ and *dnmt3ab*^−/−^ ovaries at 60 dah. (**q**) TUNEL-positive germ cells in the median section of the ovaries (part2) (*n* = 5 for each genotype). Results are presented as mean ± SD. Different letters above the error bar indicate statistical differences at *p* < 0.05 as determined by one-way ANOVA followed by Tukey’s post hoc test. dah, days after hatching. Scale bar, 10 μm. OC, oocytes; OG, oogonia. The white arrow indicated co-localization of Vasa and TUNEL signals.

Germ cells in the ovaries of *dnmt3aa*^−/−^ fish were significantly decreased. In order to further evaluate the effect of *dnmt3aa* and *dnmt3ab* mutation on the ovary development of Nile tilapia, the total and apoptotic germ cells were examined in WT, *dnmt3aa*^−/−^ and *dnmt3ab*^−/−^ fish by Vasa (germ cells marker) and TUNEL immunofluorescence co-staining. (Figure 5a–o). The number of Vasa-positive cells in *dnmt3aa*^−/−^ ovaries was significantly reduced compared with that of WT fish, but there was no difference between *dnmt3ab*^−/−^ and WT fish (Figure 5p). TUNEL assay showed that there was a large number of germ cells in apoptosis in *dnmt3aa*^−/−^ ovaries (Figure 5j), but there was no obvious germ cell apoptosis in ovaries of the *dnmt3ab*^−/−^ and WT fish (Figure 5e,o). Statistical analysis showed that the number of apoptotic germ cells in the *dnmt3aa*^−/−^ ovaries was significantly increased compared with that in the *dnmt3ab*^−/−^ and WT ovaries (Figure 5q). The expression of Cyp19a1a, the key enzyme of estrogen synthesis, was further examined and the positive signals were still observed in the *dnmt3aa*^−/−^ ovaries (Supplementary Figure S4). It is worth noting that a significant reduction in germ cells in the ovaries was also observed in the F0 generation mutants with a high mutation rate (75%) at another *dnmt3aa* target site (Supplementary Figure S5).

**Figure 6:**
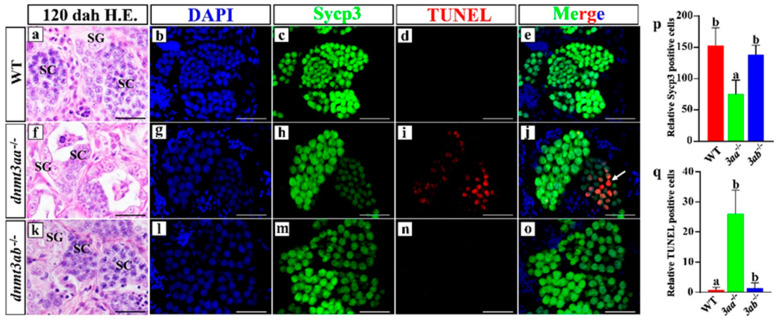
Apoptosis detection in WT, *dnmt3aa*^−/−^ and *dnmt3ab*^−/−^ testes at 120 dah. Increased apoptosis of spermatocytes in the testes of *dnmt3aa*^−/−^ fish at 120 dah. (**a**,**f**,**k**) Histological analyses of germ cells by H&E staining. (**b**,**g**,**l**) Nuclei were counterstained with DAPI. (**c**,**h**,**m**) Green fluorescence represents the Sycp3 signals. (**d**,**i**,**n**) Red fluorescence represents the TUNEL-positive signals. (**e**,**j**,**o**) Co-localization of some Sycp3 and TUNEL signals as indicated by orange color in Nile tilapia testes. (**p**) Sycp3-positive cell count of WT, *dnmt3aa*^−/−^ and *dnmt3ab*^−/−^ testes at 120 dah. (**q**) TUNEL-positive cells in the entire median section of the testes (*n* = 5 for each genotype). Results are presented as mean ± SD. Different letters above the error bar indicate statistical differences at *p* < 0.05 as determined by one-way ANOVA followed by Tukey’s post hoc test. dah, days after hatching. Scale bar, 10 μm. SC, spermatocytes; SG, spermatogonia. The white arrow indicated co-localization of Sycp3 and TUNEL signals.

Similar to the situation observed in ovaries, the number of spermatocytes in the testes of *dnmt3aa*^−/−^ fish decreased significantly at 120 dah. To further evaluate the effect of *dnmt3aa* and *dnmt3ab* mutation on the testis development of Nile tilapia, the total and apoptotic spermatocytes in WT, *dnmt3aa*^−/−^ and *dnmt3ab*^−/−^ fish were examined by Sycp3 (spermatocyte marker) and TUNEL immunofluorescence co-staining (Figure 6a–o). Statistical analysis showed that the number of Sycp3-positive cells in *dnmt3aa*^−/−^ fish was significantly lower than that of WT fish, while no significant difference in the number of Sycp3-positive cells was observed in *dnmt3ab*^−/−^ and WT fish (Figure 6p). A large number of apoptotic spermatocytes, significantly higher than that of WT and *dnmt3ab*^−/−^ fish, was observed in *dnmt3aa*^−/−^ fish (Figure 6j,q).

### 2.6. Sperm Quality of WT, Dnmt3aa^−/−^ and Dnmt3ab^−/−^ XY Fish

**Figure 7:**
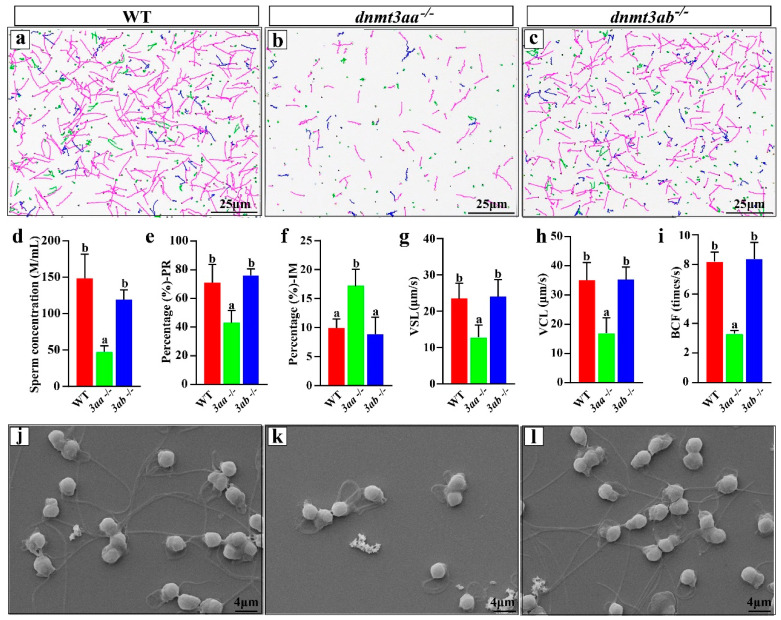
Sperm quality analyses of WT, *dnmt3aa*^−/−^ and *dnmt3ab*^−/−^ XY fish at 240 dah. (**a**–**c**) The tracks of motile sperm from WT, *dnmt3aa*^−/−^ and *dnmt3ab*^−/−^ XY fish. Pink, blue and green present grade A, B, C sperm, respectively. (**d**–**i**) The physiological characteristics of WT, *dnmt3aa*^−/−^ and *dnmt3ab*^−/−^ sperm (*n* = 5 for each genotype). PR, progressive sperm; IM, immotile sperm; VSL, straight linear velocity; VCL, curvilinear velocity; BCF, beat frequency of sperm flagella. Results are presented as mean ± SD. Different letters above the error bar indicate statistical differences at *p* < 0.05 as determined by one-way ANOVA followed by Tukey’s post hoc test. (**j**–**l**) Sperm morphology examination by scanning electron microscope.

Semen was obtained from the mature WT, *dnmt3aa*^−/−^ and *dnmt3ab*^−/−^ mutants by in vitro extrusion at 240 dah, and analyzed with a computer-assisted sperm analyzer after 1:10 dilution. The sperm from *dnmt3aa*^−/−^ mutants displayed lower activity compared with those from the WT and *dnmt3ab*^−/−^ fish (Figure 7a–c). In addition, the sperm concentration of *dnmt3aa*^−/−^ mutants was significantly lower than that of WT and *dnmt3ab*^−/−^ fish (Figure 7d). Further analysis showed that the percentage of progressive sperm in *dnmt3aa*^−/−^ fish was significantly lower than that in WT and *dnmt3ab*^−/−^ fish (Figure 7e), and the proportion of immotile sperm was significantly higher than that in WT and *dnmt3ab*^−/−^ fish (Figure 7f). Furthermore, the VSL (straight linear velocity) (Figure 7g), VCL (curvilinear velocity) (Figure 7h) and BCF (beat frequency of sperm flagella) (Figure 7i) of sperm from the *dnmt3aa*^−/−^ fish were significantly lower than those of the WT and *dnmt3ab*^−/−^ fish. Morphologically, similar to the WT sperm, the sperm from the *dnmt3ab*^−/−^ mutants were characterized by a straight and long tail, while the sperm from *dnmt3aa*^−/−^ mutants consisted of some abnormal spermatozoa with a curly and short tail from Papanicolaou staining and scanning electron microscope analysis (Figure 7j–l, Supplementary Figure S6).

### 2.7. Apoptosis Gene Expression and Compensatory Expression of Dnmt Family Genes in Dnmt3aa^−/−^ and Dnmt3ab^−/−^ Gonads

**Figure 8:**
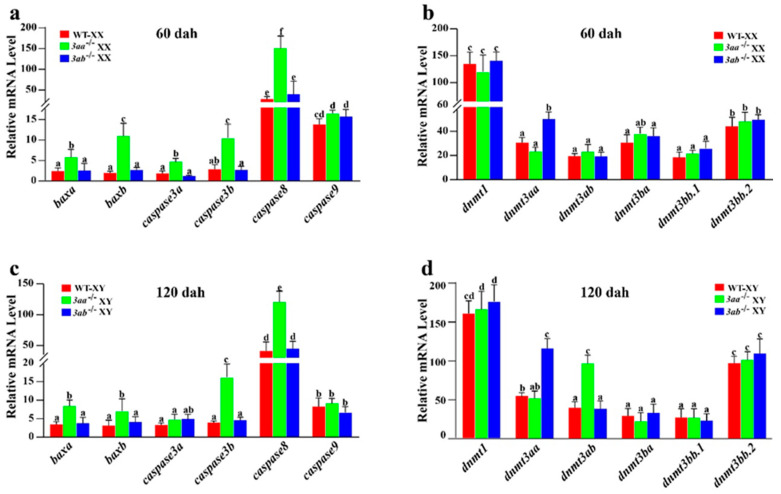
Detection of apoptosis genes and analysis of compensatory expression of *dnmt* family genes in *dnmt3aa*^−/−^ and *dnmt3ab*^−/−^ fish at 60 dah (**a**,**b**) and 120 dah (**c**,**d**). WT, wild type. The reference gene *gapdh* was used to normalize the expression values. Results are presented as mean ± SD. Different letters above the error bar indicate statistical differences at *p* < 0.05 as determined by one-way ANOVA followed by Tukey’s post hoc test. dah, days after hatching.

As mentioned above, there was a large number of germ cells in apoptosis in the gonads of *dnmt3aa*^−/−^ fish. Thus, the expression of apoptosis genes in 60 dah XX and 120 dah XY gonads was further analyzed by quantitative real-time PCR (qRT-PCR). The results showed that the expression of apoptosis genes *baxa*, *baxb*, *caspase3a*, *caspase3b* and *caspase8* was significantly increased in *dnmt3aa*^−/−^ ovaries at 60 dah, but there was no significant difference between *dnmt3ab*^−/−^ and WT ovaries (Figure 8a). Gonads of *dnmt3aa* and *dnmt3ab* homozygous mutants were analyzed for gene compensatory expression. The homozygous mutation of *dnmt3ab* caused a compensatory increase in *dnmt3aa* expression at 60 dah. However, there was no significant difference in the expression of other *dnmts* between the mutants and WT fish (Figure 8b). At 120 dah, the expression of apoptosis genes *baxa*, *baxb*, *caspase3b* and *caspase8* in *dnmt3aa*^−/−^ males was significantly increased (Figure 8c). Interestingly, *dnmt3aa* and *dnmt3ab* were found to compensate for each other in the homozygous mutant males at 120 dah (Figure 8d).

### 2.8. The Global DNA Methylation Level of WT, Dnmt3aa^−/−^ and Dnmt3ab^−/−^ Gonads

**Figure 9:**
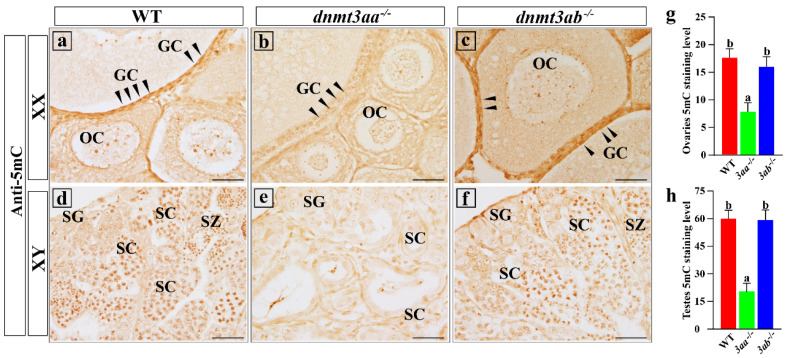
5-methylcytosine (5-mC) staining of WT, *dnmt3aa*^−/−^ and *dnmt3ab*^−/−^ gonads at 120 dah. (**a**–**c**) 5-mC staining of WT, *dnmt3aa*^−/−^ and *dnmt3ab*^−/−^ ovaries. Positive signals were observed in the nuclei of oocytes and granulosa cells. (**d**–**f**) 5-mC staining of WT, *dnmt3aa*^−/−^ and *dnmt3ab*^−/−^ testes. Positive signals were observed in spermatogonia, spermatocytes and spermatozoa. The positive signals correspond to the brownish color. (**g**) Statistical analysis of relative 5-mC staining level in WT, *dnmt3aa*^−/−^ and *dnmt3ab*^−/−^ ovaries. (**h**) Statistical analysis of relative 5-mC staining level in WT, *dnmt3aa*^−/−^ and *dnmt3ab*^−/−^ testes (*n* = 5, and five sections were counted per sample). The IHC positive signals were quantified using image J software according to the instructions. Results are presented as mean ± SD. Different letters above the error bar indicate statistical differences at *p* < 0.05 as determined by one-way ANOVA followed by Tukey’s post hoc test. OC, oocytes; GC, granulosa cells; SC, spermatocytes; SG, spermatogonia; SZ, spermatozoa. Scale bar, 10 μm. The black arrow indicated 5-mC-positive signals in granulosa cells.

Immunoreactive signals of 5-methylcytosine (5-mC) were predominantly present in the nuclei of oocytes and granulosa cells in ovaries (Figure 9a–c) and spermatogonia, spermatocytes and spermatozoa in testes (Figure 9d–f). The immunoreactive signals were strong in spermatocytes and spermatozoa but weak in spermatogonia (Figure 9d–f). Statistical analysis showed that the 5-mC levels in ovaries and testes of *dnmt3aa*^−/−^ fish were significantly lower than those of WT and *dnmt3ab*^−/−^ fish. There were no significant differences in the 5-mC levels in ovaries and testes between *dnmt3ab*^−/−^ and WT fish (Figure 9g,h). Further analysis showed that the 5-mC levels of granulosa cells in ovaries and spermatogonia, spermatocytes and spermatozoa in testes of *dnmt3aa*^−/−^ fish were significantly lower than those of *dnmt3ab*^−/−^ and WT fish (Supplementary Figure S7). These results showed that the mutation of *dnmt3aa* significantly reduced the 5-mC levels in Nile tilapia ovaries and testes.

## 3. Discussion

DNA methylation, mediated by *dnmts*, is required for proper embryonic development and for the formation of mature functional germ cells [37]. *Dnmt3a*, one of the *dnmts*, is responsible for de novo methylation of mammalian germ cells and plays crucial roles in mammalian gonad development [17,38,39,40]. In teleosts, there are two *Dnmt3a* paralogs, *dnmt3aa* and *dnmt3ab*, due to the third round of genome duplication [21]. The expression patterns and roles of *dnmt3aa* and *dnmt3ab* in gonadal development remain poorly understood in teleosts. In the present study, the expression patterns of *dnmt3aa* and *dnmt3ab* were examined at different development stages of gonads in Nile tilapia. Homozygous mutation of *dnmt3aa* and *dnmt3ab* was established and gonadal phenotypes and possible mechanisms were analyzed and discussed.

### 3.1. Different Expression Patterns of Dnmt3aa and Dnmt3ab in Gonads Indicate the Sub-Functionalization in Teleosts

Gene expression patterns are important aspects of gene regulation and function analysis. The analysis of *Dnmt3a* expression patterns in gonads is mainly focused on mammals [19,41], but rarely reported in fish. In humans (*Homo sapiens*), *Dnmt3a* mRNA expression is detected in the ovarian follicles from primordial to secondary follicles, granulosa cells, and germinal vesicle (GV) and metaphase II (MII) oocytes [42]. In addition, *Dnmt3a* is expressed in human spermatogenic cells, including spermatogonia cells, primary spermatocytes, secondary spermatocytes and round spermatids [43]. In rhesus monkeys (*Macaca mulatto*), *Dnmt3a* mRNA is expressed in follicles at different developmental stages [44]. In female mice, the Dnmt3a protein is localized around the nucleus of GV oocytes and in the cytoplasm of the MII oocytes [45]. In male mice, *Dnmt3a* is expressed at mRNA and protein levels in male germ cells, at a high level in type A spermatogonia, slightly decreased in type B spermatogonia, and further decreased in preleptotene and pachytene spermatocytes [13,14]. In general, *Dnmt3a* is expressed in mammalian follicles and granulosa cells of ovaries and spermatogenic cells of testes. There are few studies on the cellular localization of *dnmt3aa* and *dnmt3ab* in teleosts except for a report describing immunoreactive signals for Dnmt3a (Dnmt3aa and Dnmt3ab) in male germ cells, particularly in spermatocytes in ricefield eels [25]. In this study, we comprehensively studied the expression patterns and their cellular localization of *dnmt3aa* and *dnmt3ab* in the ovaries and testes of Nile tilapia. *Dnmt3aa* and *dnmt3ab* displayed sexually dimorphic expression profiles in developing gonads, with a higher expression in testes than in ovaries. Furthermore, higher expression was observed for *dnmt3aa* than *dnmt3ab* at all the time points examined, suggesting that *dnmt3aa* may play more important roles in gonadal development than *dnmt3ab*. In Nile tilapia, *dnmt3aa* was highly expressed in oogonia, phase I and II oocytes and granulosa cells, while *dnmt3ab* was mainly expressed in granulosa cells of the ovaries. Both were highly expressed in spermatogonia and spermatocytes of the testes. These results demonstrated that the expression of *dnmt3aa* and *dnmt3ab* in Nile tilapia was basically consistent with that in mammals. Interestingly, in ovaries, *dnmt3aa* was expressed in both germ cells (oogonia and oocytes) and somatic cells (granulosa cells), while *dnmt3ab* was only expressed in somatic cells (granulosa cells). The different expression patterns of *dnmts* in gonads are probably essential for the acquisition of a sex-specific methylation pattern [46]. The different expression patterns of *dnmt3aa* and *dnmt3ab* in the gonads of Nile tilapia suggest that they may play different roles in the gonadal development of Nile tilapia.

### 3.2. Homozygous Mutants of Dnmt3aa or Dnmt3ab Are Viable in Nile Tilapia

The roles of *Dnmt3a* in early development and gametogenesis in mammals have been investigated by gene targeting disruption. Although *Dnmt3a^−/−^* mice develop to term and appear to be normal at birth, most homozygous mutant mice become runted and die at about 4 weeks of age [47]. These results indicate that the deficiency of *Dnmt3a* in mice is fatal. However, recent studies on zebrafish have shown that the homozygous mutants of *dnmt3aa* or *dnmt3ab* are viable. *Dnmt3aa* and *dnmt3ab* are demonstrated to have essential and non-overlapped functions in modulating behavioral control, and *dnmt3aa* or *dnmt3ab* mutation fish display anxiety symptoms [30]. In addition, some groups have demonstrated the roles of *dnmt3aa* or *dnmt3ab* in regulating developmental thermal plasticity, and the phenotypic effects of *dnmt3aa* and *dnmt3ab* are additive [31]. In this study, the growth and survival of the homozygous mutants of *dnmt3aa* and *dnmt3ab* were grossly normal compared with the WT fish. Different spatial–temporal expression profiles of *dnmt3aa* and *dnmt3ab*, the up-regulation of *dnmt3ab* in *dnmt3aa*^−/−^ gonads and the up-regulation of *dnmt3aa* in *dnmt3ab*^−/−^ gonads were observed. These results indicate the sub-functionalization and compensation of *dnmt3aa* and *dnmt3ab* in Nile tilapia, which might be possible reasons for the survival of the mutants. As suggested in a previous study [48], the existence of duplicate genes probably increased the survival opportunities of the species. Our study provided another example for this notion.

### 3.3. Deficiency of Dnmt3aa, but Not Dnmt3ab, Results in Decrease in GSI, Reduction in Germ Cells and Decline in Sperm Quality in Nile Tilapia

In mammals, *Dnmt3a* is essential for both male and female germ lines’ de novo methylation [5,17,49]. During follicular development after birth, DNA methylation plays important roles in the regulation of gene expression in oocytes. DNA methylation also plays crucial roles in normal spermatogenesis. Previous studies have demonstrated that male germ cells in mice have a highly distinct epigenetic pattern, characterized by a unique genome-wide pattern of DNA methylation. During spermatogenesis, global remethylation is established from spermatogonia to spermatocytes, and continues in the round spermatids and spermatozoa [50,51]. It has been reported that abnormal DNA methylation in spermatogenic cells due to genetic failure, environmental factors and disturbed expression of the *dnmts* may lead to spermatogenic impairments [52,53,54]. Indeed, azoospermia studies in humans have shown that significant changes in *dnmts* expression and DNA methylation levels in spermatogenic cells may lead to male infertility [55]. In this study, mutants of *dnmt3aa* showed atrophy and degeneration of ovaries and more transparent testes, a lower GSI and a significant decrease in oocytes and spermatocytes, while mutants of *dnmt3ab* showed normal development of ovaries and testes. Our results demonstrate that *dnmt3aa*, but not *dnmt3ab*, plays important roles in gonadal development and gamogenesis.

In humans, low sperm motility is associated with decreased sperm methylation [56]. Compared with WT and *dnmt3ab*^−/−^ fish, the *dnmt3aa*^−/−^ mutants displayed a significantly lower sperm concentration and significantly reduced progressive sperm proportion. The VSL, VCL and BCF in *dnmt3aa*^−/−^ mutants were significantly lower than those of the WT and *dnmt3ab*^−/−^ fish. Further observation and analysis showed that compared with the WT, tails of some sperm in *dnmt3aa*^−/−^ fish were shorter and curved, which might account for the decrease in sperm motility.

### 3.4. Low Global DNA Methylation Results in Germ Cell Apoptosis and Abnormal Phenotype in Gonads of Dnmt3aa^−/−^ Mutants

DNA methylation is essential for the normal development of cells, and abnormal DNA methylation can result in cell apoptosis [57]. Previous reports have demonstrated that the conditional knockout of *Dnmt3a* in mice results in a significant reduction in DNA methylation in oocytes [49,58,59]. In this study, the mutation of *dnmt3aa* significantly reduced the global DNA methylation levels in Nile tilapia ovaries and testes. Further analysis showed that the 5-mC levels of granulosa cells in ovaries and spermatogonia, spermatocytes and spermatozoa in testes of *dnmt3aa*^−/−^ fish were significantly lower than those of *dnmt3ab*^−/−^ and WT fish. Our results indicate that *dnmt3aa* is essential for the correct establishment of DNA methylation patterns of gonads in female and male Nile tilapia. The deficiency of *dnmt3aa* results in a significant reduction in DNA methylation, which induces apoptotic gene expression and germ cell apoptosis. In this study, a significant increase in apoptosis signals in oocytes and spermatocytes was observed in *dnmt3aa*^−/−^ mutants and the expressions of apoptotic genes were significantly increased. The apoptosis of germ cells resulted in a decrease in GSI, reduction in germ cells and gonadal degeneration in female and male mutants.

In female mice, conditional knockout of *Dnmt3a* in oocytes results in the failure of DNA methylation establishment in oocytes, loss of maternal imprints and death of offspring in the uterus [5,17,39]. However, mouse oocytes with *Dnmt3a* conditional knockout are capable of being fertilized, different from the phenotype observed in Nile tilapia in which global mutation of *dnmt3aa* caused oocyte apoptosis and ovarian degeneration. The discrepancy might be attributed to the following reasons. The oocyte does not develop in isolation but is instead highly dependent on surrounding granulosa cells of the intact ovarian follicle. The process of oocyte development is supported by granulosa cells, and bidirectional communication between oocytes and granulosa cells is important for the development and maturation of the oocytes [60,61,62]. Granulosa cells undergo dynamic DNA methylation changes to repress or activate the genes required for their proliferation and differentiation during follicular development [63,64]. Correctly established DNA methylation in granulosa cells is important for the regulation of the expression of genes related to follicular development [65]. Changing *Dnmt3a* gene expression in granulosa cells causes impaired oocyte maturation in the GV and MII oocytes and subsequent abnormal embryonic development [20,66]. In this study, the mutation of *dnmt3aa* resulted in a significant reduction in the methylation level of ovarian granulosa cells. The significant decrease in the 5-mC level of granulosa cells affected the development of oocytes and resulted in oocyte apoptosis in Nile tilapia. In addition, global mutation of *dnmt3aa* might also influence estrogen synthesis via the hypothalamic–pituitary–gonadal axis and result in defects in vitellogenesis, which, in turn, may result in oocyte apoptosis. It is worth noting that in zebrafish, medaka (*Oryzias latipes*) and Nile tilapia, a significant decrease in ovarian germ cells led to female to male sex reversal [35,67,68,69]. In this study, positive signals of Cyp19a1a were observed in *dnmt3aa*^−/−^ ovaries, indicating the mutants were still female. In fact, a small number of oocytes in the ovaries continued to develop at 120 and 240 dah. The remaining germ cells maintained the ovarian phenotype.

In male mice, germ cells without *Dnmt3a* undergo meiotic failure, impaired spermatogenesis, which results in no spermatocytes, spermatids or spermatozoa, and significantly reduced testis size [17]. However, viable sperm were produced in *dnmt3aa*^−/^^−^ fish even though the sperm concentration and motility were significantly lower than those of WT fish, which is different from the phenotype of *Dnmt3a* conditional mutation in mice. A significant up-regulation of *dnmt3ab* expression in *dnmt3aa*^−/^^−^ testes indicated that the compensatory increase in *dnmt3ab* might be one of the reasons for these differences.

## 4. Materials and Methods

### 4.1. Animal Rearing Conditions

Nile tilapia (parental fish, one year old) were raised in circulating aerated fresh water tank under natural light at 26 ± 0.5 °C. The water quality parameters were monitored daily (pH: 7.2 ± 0.5; dissolved oxygen: 6.5–7.0 mg/L). Fish were fed three times a day with commercial feed (Shengsuo, Yantai, China). All-XX progenies were obtained by crossing a pseudomale (XX male, producing sperm after hormonal sex reversal) with a normal XX female. All-XY progenies were obtained by crossing a YY super male with an XX female. Larvae and juveniles were raised under the same conditions as the parental fish. Animal experiments were conducted in accordance with the regulations of Guide for Care and Use of Laboratory Animals and were approved by the Committee of Laboratory Animal Experimentation at Southwest University, China. (No. IACUC-20181015-12, 15 October 2018).

### 4.2. Establishment of Dnmt3aa and Dnmt3ab Homozygous Mutant Lines by CRISPR/Cas9

Briefly, the gRNA of *dnmt3aa*/*dnmt3ab* and Cas9 mRNA were co-injected into one-cell-stage embryos (0–90 min after fertilization) at a final concentration of 250 and 500 ng/µL, respectively. All of the injected embryos were incubated at 26 °C. Twenty injected embryos were collected at 72 h after injection. Genomic DNA was extracted from pooled control and injected embryos and used to assess the mutations. DNA fragments spanning the target site were amplified. The mutated sequences were analyzed by restriction enzyme digestion (*Hpy* 188III for *dnmt3aa* and *Mly* I for *dnmt3ab*) and Sanger sequencing.

Heterozygous F1 offspring were obtained by F0 XY male founders mated with WT XX females. The F1 fish were genotyped by fin clip assay and the individuals with frame-shift mutations were selected. XY male and XX female siblings of F1 generation, carrying the same mutation, were mated to generate homozygous F2 mutants. The F1 mutant fish that carried 4 and 5 base-pair deletions were used for construction of F2 *dnmt3aa* and *dnmt3ab* mutants, respectively. The *dnmt3aa* and *dnmt3ab* F2 mutants were screened using restriction enzyme digestion and Sanger sequencing. The genetic sex of each fish was determined by genotyping using sex-linked marker (marker 5) as described previously [33,70].

### 4.3. Gonad Morphological and Histological Analysis

To investigate the roles of *dnmt3aa* and *dnmt3ab* in gonadal development, the gross morphology and histology of WT, *dnmt3aa*^−/−^ and *dnmt3ab*^−/−^ ovaries and testes were analyzed at 60, 120 and 240 dah. The fish were anesthetized with MS-222 (Sigma-Aldrich, St. Louis, MO, USA) and the gonad morphology of the mutants and WT fish was imaged by stereomicroscope (Leica, Bensheim, Germany) after biopsy. The body and gonad weight were measured for GSI calculating (*n* = 10 for each genotype). Then, the gonads were fixed in Bouin’s solution for 24 h at room temperature. The fixed samples were then processed as follows: serial dehydration in 70, 80 and 90% ethanol for 1.5 h each, 95% ethanol for 2 h and 100% ethanol three times for 1 h each; sequential clearance in xylene and ethanol mixture (1:1) for 30 min and xylene twice for 30 min each; and infiltration in paraffin 2 h. The samples were sectioned at 5 μm thickness using the Leica microtome (Leica Microsystems, Wetzlar, Germany). The sections were stained with hematoxylin and eosin (H&E) as described previously [71]. Photographs were taken under Olympus BX51 light microscope (Olympus, Tokyo, Japan). Sibling WT fish were used as control for phenotype analysis. Germ cells from the median sections of testes (*n* = 5 for each genotype) and different parts (1–3) of ovaries (*n* = 5 for each genotype) were counted for statistical analyses. The histological classification of the follicles and spermatogenic cells for Nile tilapia was performed according to the standards described previously [72,73].

### 4.4. Gene Expression Analyses by qRT-PCR

Gonads of the WT, *dnmt3aa*^−/−^ and *dnmt3ab*^−/−^ fish were dissected at different developmental stages (5, 30, 60, 90, 120 and 180 dah) for gene expression assay. Three parallel ovary and testis samples with different number of gonads based on the age of fish were prepared at each developmental stage. Total RNA (1.0 μg) was extracted and treated with DNase I to eliminate genomic DNA contamination, and was reverse transcribed using PrimeScript RT Master Mix Perfect Real Time Kit according to the manufacturer’s instructions (Takara, Dalian, China). qRT-PCR was performed on an ABI7500 qRT-PCR machine, according to the protocol of SYBR Premix Ex TaqTM II (Takara, Dalian, China). The relative abundance of key genes in the gonad was evaluated using the formula R = 2^−△△Ct^ [74]. The reference gene *gapdh* was used to normalize the expression values. Primer sequences used in this study are listed in Supplementary Table S1.

### 4.5. Immunofluorescence (IF), Terminal Deoxynucleotidyl Transferase-Mediated dUTP Nick End-Labeling (TUNEL) and Immunohistochemistry (IHC)

The Vasa, Cyp19a1a and Sycp3 rabbit polyclonal antibodies were prepared by our laboratory. The dilution and specificity of these antibodies have been analyzed previously [75,76,77]. For IF, Alexa Fluor 488- and 594-conjugated secondary antibodies (Invitrogen, Shanghai, China) were diluted 1:500 in blocking solution and incubated with tissue to detect the primary antibodies. The nuclei were stained by 4′,6-diamidino-2-phenylindole (DAPI) (Invitrogen, Carlsbad, CA, USA). Apoptosis of germ cells was evaluated by staining paraffin sections of WT, *dnmt3aa*^−/−^ and *dnmt3ab*^−/−^ mutant fish at 60 (ovaries) and 120 dah (testes) with in situ cell death detection kit, TMR red TUNEL system (Roche, Mannheim, Germany) according to the manufacturer’s protocol. Apoptotic germ cells in the testes (entire median section, *n* = 5 for each genotype) and ovaries (part 2, *n* = 5 for each genotype) were counted for statistical analysis. Fluorescence signals were captured by confocal microscopy (Olympus FV3000) (Olympus, Tokyo, Japan). The assessment of global DNA methylation in WT, *dnmt3aa*^−/−^ and *dnmt3ab*^−/−^ gonads was performed with immunohistochemistry using the anti-5-mC antibody (MABE146, Merck Millipore) according to previous reports [20,25]. Photographs were taken under an Olympus BX51 light microscope (Olympus, Tokyo, Japan). Finally, the positive signals were quantified using image J software (National Institutes of Health, Bethesda, MD, USA).

### 4.6. Fluorescence In Situ Hybridization (FISH)

The open reading frames of Nile tilapia *dnmt3aa* and *dnmt3ab* were amplified with specific primers (Supplementary Table S1), and the amplicons were cloned into pGEM-T Easy Vector. The sense and anti-sense RNA probes were labeled with digoxigenin (DIG) by in vitro transcription using an RNA labeling kit (Roche, Mannheim, Germany). The gonads of fish were sampled at indicated time. The fish gonads were fixed in 4% paraformaldehyde in phosphate-buffered saline (PBS) and processed for serial paraffin sectioning at 5 μm thickness. The sections were deparaffinized, rehydrated and digested with proteinase K (4 μg/mL; Roche, Mannheim, Germany) at 37 °C for 15 min, followed by hybridization with DIG-labeled RNA probes at 60 °C overnight. The sections were washed with 50% formamide/2 × SSC for 30 min, 2 × SSC for 20 min and 0.2 × SSC for 20 min. The slides were incubated for 30 min at room temperature in a humidified chamber with Anti-DIG-POD (Roche, Mannheim, Germany) diluted by 200 times in DIG2 buffer. Then, the sections were washed with DIG1 buffer. After washing, the TSA Plus Fluorescein System (PerkinElmer, Boston, MA, USA) was used for the amplification of hybridization signals. The nuclei were stained by DAPI (Invitrogen, Carlsbad, CA, USA) staining. Fluorescence signals were captured by confocal microscopy (Olympus FV3000) (Olympus, Tokyo, Japan).

### 4.7. Sperm Mobility Analysis

Sperm concentration, sperm motility (%), curvilinear velocity (VCL), straight line velocity (VSL) and beat frequency of sperm flagella (BCF) were examined by computer assisted sperm analysis using the Sperm Quality Analyzer according to the manufacturer’s instructions (Zoneking Software, China). Briefly, semen collected from WT, *dnmt3aa*^−/−^ and *dnmt3ab*^−/−^ XY fish (*n* = 5 for each genotype) at 240 dah was diluted with PBS at 1:10. After sperm activation, one drop of semen was dripped into the counting pool of the sperm counting board, and placed on the operating platform of a Leica DM500 light microscope (Leica, Bensheim, Germany). All parameters were collected according to instrument instructions.

### 4.8. Sperm Papanicolaou Staining and Scanning Electron Microscope Analysis

Semen from WT, *dnmt3aa*^−/−^ and *dnmt3ab*^−/−^ fish (*n* = 5 for each genotype) was collected by in vivo extrusion and then 1 μL of drained semen mixed with 9 μL of double distilled water was applied to clean slides, which were dried naturally and then stained by Papanicolaou staining. Photographs were taken under Olympus BX51 light microscope (Olympus, Tokyo, Japan). To further examine sperm morphology, semen was collected for scanning electron microscope analysis. In brief, sperm specimens were pre-fixed using 2.5% glutaraldehyde, rinsed three times with PBS (pH 7.2) and dehydrated in ascending graded ethanol. Then, the dehydrated samples were put into a drying basket and dried with critical point dryer. The surface of the dried samples was treated with electric conduction, and the specimens were observed under a Zeiss Evo LS10 (Zeiss, Oberkochen, Germany) scanning electron microscope.

### 4.9. Data Analyses

All data are presented as mean ± SD from at least three independent experiments. Different letters above the error bar indicate statistical differences at *p* < 0.05 as determined by one-way ANOVA followed by Tukey’s post hoc test. Statistics analyses were performed using GraphPad Prism 8 software package (GraphPad Software, La Jolla, CA, USA).

## 5. Conclusions

In this study, it was demonstrated that both *dnmt3aa* and *dnmt3ab* displayed sexually dimorphic expression in developing gonads. *Dnmt3aa* was highly expressed in oogonia, phase I and II oocytes and granulosa cells in ovaries and spermatogonia and spermatocytes in testes, while *dnmt3ab* was mainly expressed in ovarian granulosa cells and testicular spermatocytes. The mutation of *dnmt3aa* resulted in a lower GSI, increased apoptosis of oocytes and spermatocytes and significantly reduced sperm quality, while no obvious phenotype was observed in *dnmt3ab* homozygous mutants. The level of 5-mC in *dnmt3aa*^−/−^ mutant testes and ovaries decreased significantly, while there was no difference between *dnmt3ab*^−/−^ mutants and WT. Our results suggest that *dnmt3aa*, not *dnmt3ab*, plays important roles in maintaining normal gametogenesis in teleosts. Our results enrich the understanding of the function of DNA methyltransferase in gonads of non-mammalian vertebrates.

## Data Availability

All important data is included in the manuscript.

## Supplementary Materials

The following are available online at https://www.mdpi.com/article/10.3390/ijms221810170/s1.
