# Supplementary material for: Dnmt3aa but Not Dnmt3ab Is Required for Maintenance of Gametogenesis in Nile Tilapia (Oreochromis niloticus)
Source: Int J Mol Sci. 2021 Sep 21;22(18):10170. doi: 10.3390/ijms221810170 (PMC8469005; doi:10.3390/ijms221810170)
Supplement: Supplementary file 1 [file ijms-22-10170-s001.zip › ijms-1365430-supplementary.pdf]

Supplementary Figure S1

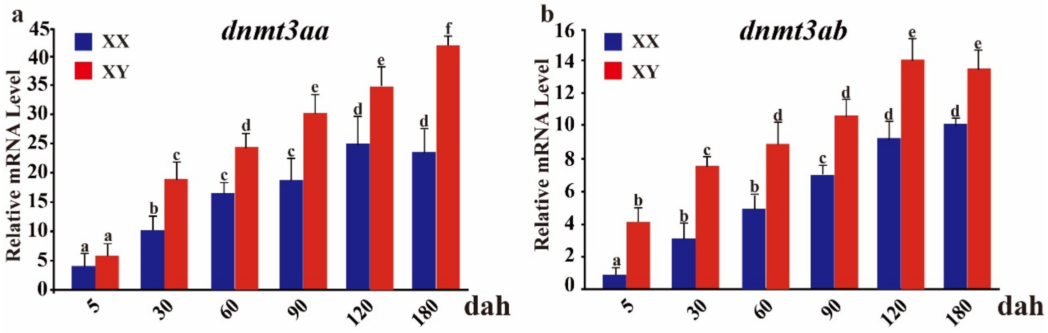

Supplementary Figure S1. (**a-b**) Ontogenetic expression of *dnmt3aa* and *dnmt3ab* by qRT-PCR. Results were presented as mean ± SD. *gapdh* was selected as the internal control. Different letters above the error bar indicate statistical differences at $p < 0.05$, as determined by one-way ANOVA followed by Tukey's post-hoc test. dah, day after hatching.

Supplementary Figure S2

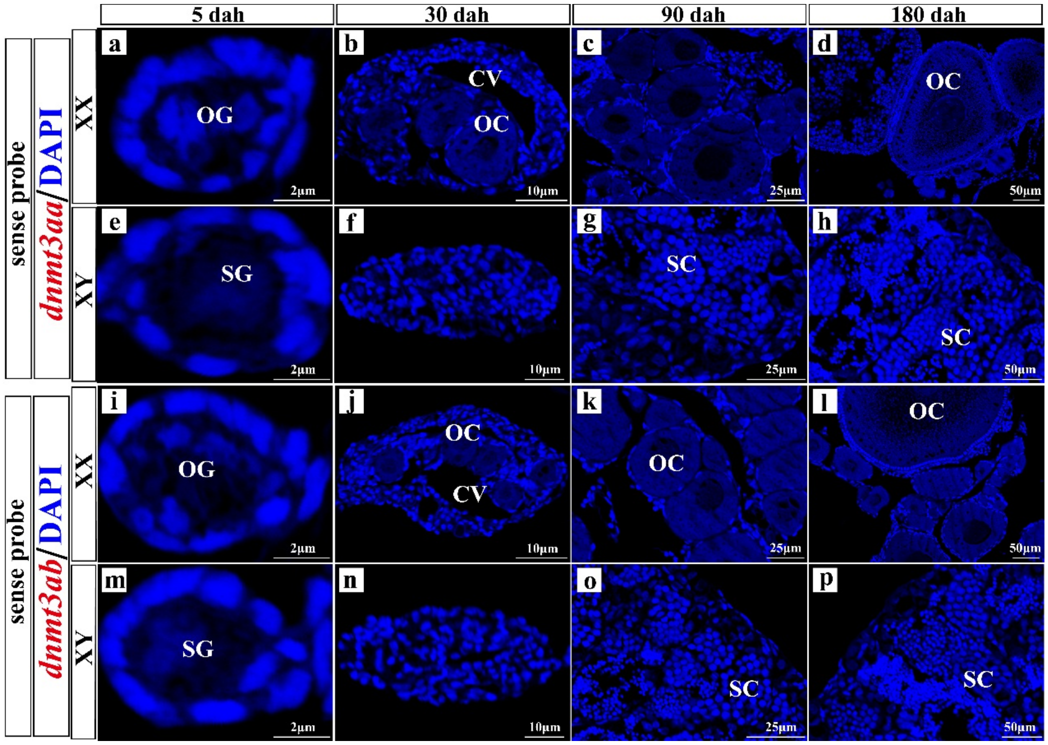

Supplementary Figure S2. (**a-p**) Cellular localization of *dnmt3aa* and *dnmt3ab* in tilapia testes and ovaries at different developmental stages by FISH. No signal for *dnmt3aa* and *dnmt3ab*

were detectable in the ovaries and testes using the sense probe. dah, day after hatching.

Supplementary Figure S3

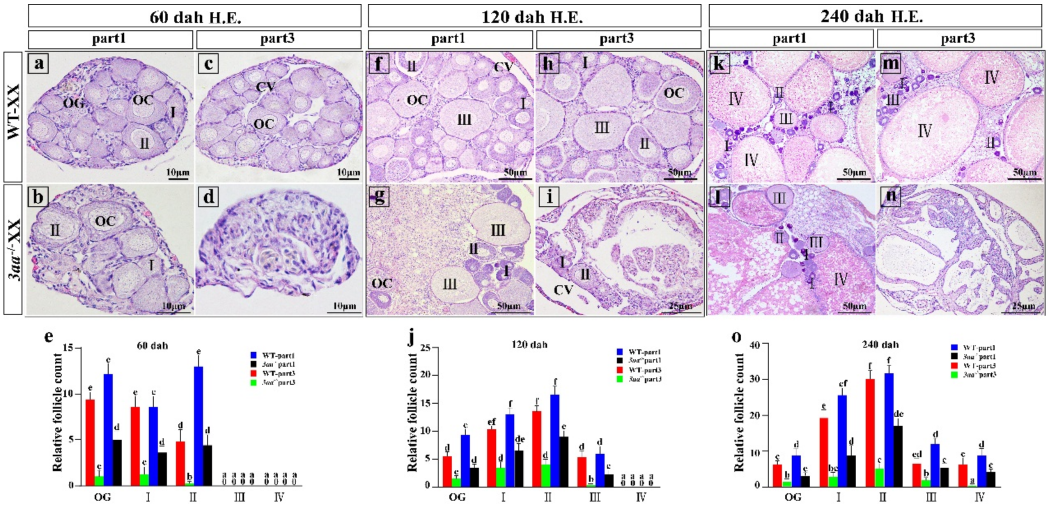

Supplementary Figure S3. (**a-d**, **f-i**, **k-n**) Histological observation WT and *dnmt3aa*$^{-/-}$ ovaries of part1 and part3 at 60, 120 and 240 dah. (**e**, **j**, **o**) Statistical analysis the number of WT and *dnmt3aa*$^{-/-}$ part1 and part3 ovarian follicles at 60, 120 and 240 dah. Results were presented as mean ± SD. Different letters above the error bar indicate statistical differences at $p < 0.05$ as determined by one-way ANOVA followed by Tukey's post-hoc test. dah, day after hatching.

Supplementary Figure S4

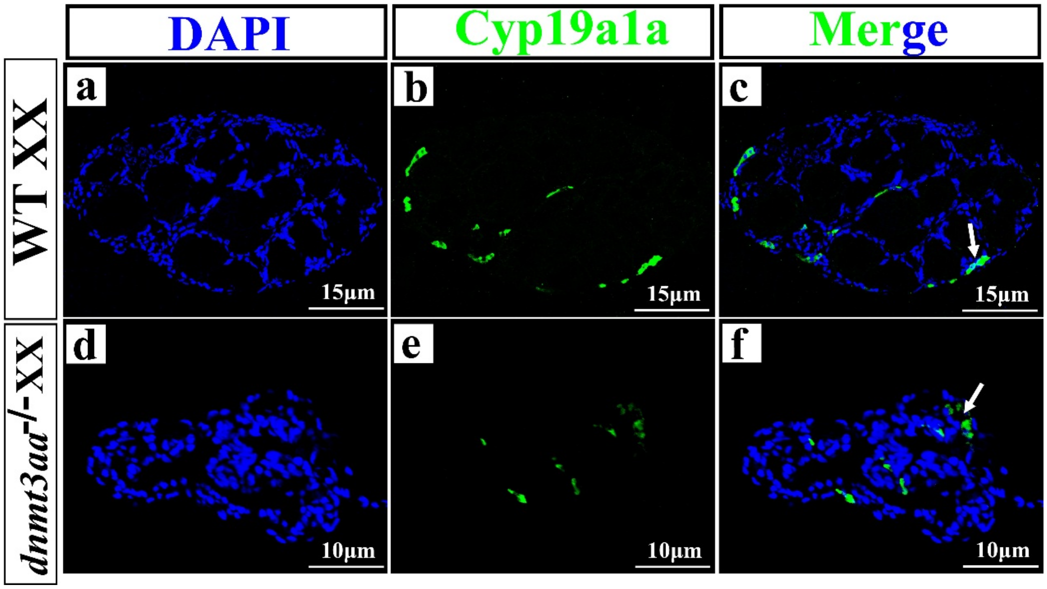

Supplementary Figure S4. Expression analysis of *cyp19a1a* (female pathway key gene) in WT (**a-c**) and *dnmt3aa*$^{-/-}$ ovaries (**d-f**). Green fluorescence represents the Cyp19a1a signals (white arrows). Blue fluorescence represents the DAPI signals.

Supplementary Figure S5

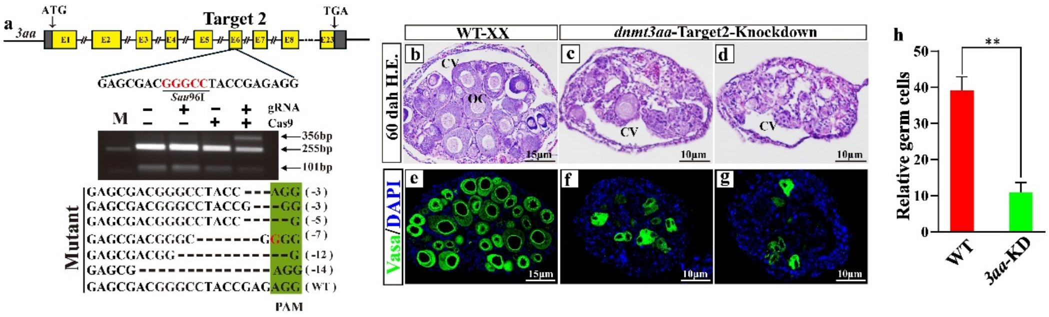

Supplementary Figure S5. Knockdown *dnmt3aa* by CRISPR/Cas9 in another target site. (**a**) Gene structures of *dnmt3aa* showing the second target site and the *Sau 96*I restriction site. The Cas9 mRNA and gRNA were added as indicated. Sanger sequencing results from the uncleaved bands were listed. The PAM was marked in light green. (**b-d**) Histological analysis ovaries of WT and *dnmt3aa* highly efficient knockdown females. (**e-g**) Analysis of Vasa (germ cells marker) expression in WT and *dnmt3aa* knockdown ovaries by IF. Green fluorescence represents the Vasa signals. Blue fluorescence represents the DAPI signals. (**h**) Statistical analysis the germ cells number of WT and *dnmt3aa* knockdown fish (n = 5). Results were presented as mean ± SD. ** p< 0.01.

Supplementary Figure S6

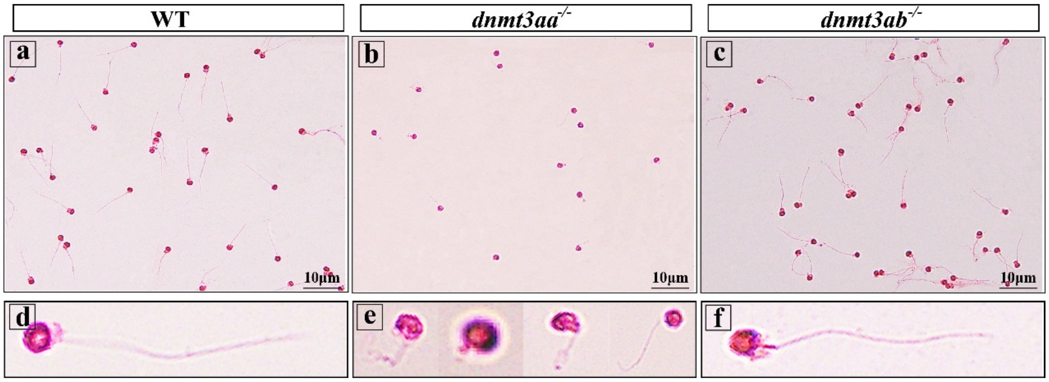

Supplementary Figure S6. Papanicolaou staining analysis of WT, *dnmt3aa*$^{-/-}$ and *dnmt3ab*$^{-/-}$ sperms. d, e, f are the amplification of a, b and c, respectively.

Supplementary Figure S7

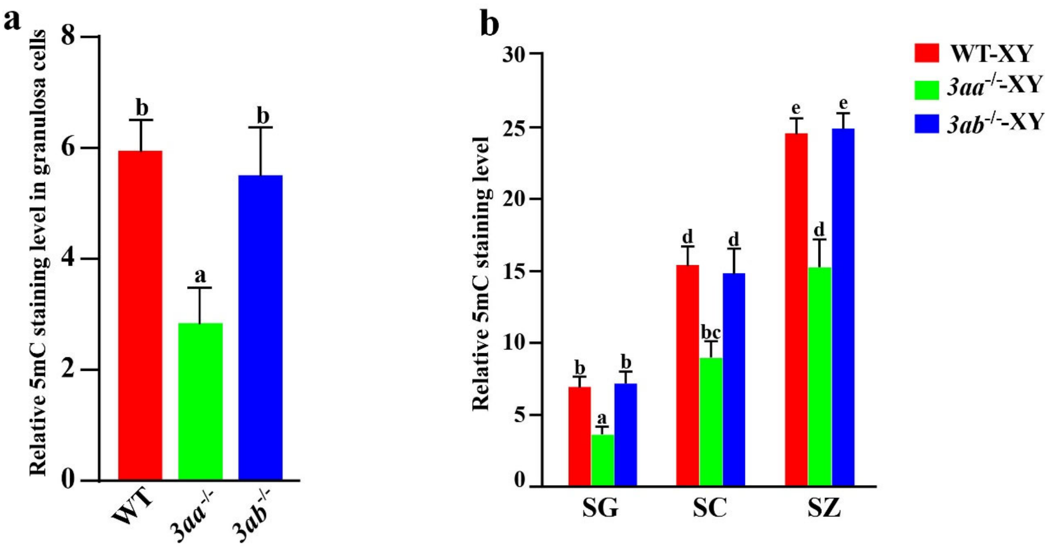

Supplementary Figure S7. (**a**) Statistical analysis relative 5-mC staining level of granulosa cells in WT, *dnmt3aa*$^{-/-}$ and *dnmt3ab*$^{-/-}$ ovaries at 120 dah. (**b**) Statistical analysis relative 5-mC staining level of WT, *dnmt3aa*$^{-/-}$ and *dnmt3ab*$^{-/-}$ spermatogonia, spermatocytes and spermatozoa in testes at 120 dah. Results were presented as mean ± SD. Different letters above the error bar indicate statistical differences at $p < 0.05$ as determined by one-way ANOVA followed by Tukey's post-hoc test. SG, spermatogonia; SC, spermatocytes; SZ, spermatozoa. dah, day after hatching.

Supplementary Table S1. All primer sequences used in this study.

**Primer name**	**Sequence (5'-3')**	**Purpose**
gRNA-*dnmt3aa*-target 1-F	TAATACGACTCACTATAGGCAGACCA GGACAGTCCAGGTTTTAGAGCTAGAA	CRISPR/Cas9
gRNA-*dnmt3aa*-target 2-F	TAATACGACTCACTATAGGAGCGA CGGGCCTACCGAGGTTTTAGAGCTAGAA	
gRNA-*dnmt3ab*-target 1-F	TAATACGACTCACTATAGGAGAGAA CAATACTGGACTGTTTTAGAGCTAGAA	
gRNA-R	AGCACCGACTCGGTGCCAC	
dnmt3aa-KO-T1-detect-F	CCTGAAGAGCTTCTTAGCCACA	Mutant screening
dnmt3aa-KO-T1-detect-R	ATGCATGTTTAACAAACCTCTGC	
dnmt3ab-KO-detect-F	TGTCTCATTGTCCACTCAACCA	
dnmt3ab-KO-detect-R	TGTTGAATCAGACCTAATCACTGC	
dnmt3aa-KO-T2-detect-F	AACCTGCCAGCATGTACTCG	
dnmt3aa-KO-T2-detect-R	TGTTGTTGTGAAGGGGGAGG	
dnmt3aa-RT-PCR-F	CAGTCCAGAGGAGGGG	RT-PCR
dnmt3aa-RT-PCR-R	TCTCCCCTGGCGACTGGCTCGG	
dnmt3ab-RT-PCR-F	TGGACTCGGACCTGAT	
dnmt3ab-RT-PCR-R	CCTCCGCACGGCAAGAGGAAGG	
gapdh-F	AAGCTCATTTCCTGGTAT	
gapdh-R	CCTTTGCTGATTTCCTTG	
dnmt3aa-ISH-F	CCCAGACACCAGAGAACGAC	ISH
dnmt3aa-ISH-R	TGTTGTTGTGAAGGGGGAGG	
dnmt3ab-ISH-F	TCCACCAAAGCTTTACCCCC	
dnmt3ab-ISH-R	CCTGTTCATGCCAGGAAGGT	
dnmt1-qPCR-F	TGGCTCCCACGTTGATGAC	qRT-PCR
dnmt1-qPCR-R	AATTTGCCTTGCTCCTCCGT	
dnmt3aa-qPCR-F	TTGAGCCGGCCAAGTTGTAT	

dnmt3aa-qPCR-R	CATGCCGACAGTGATGGAGT	
dnmt3ab-qPCR-F	TCCACCAAAGCTTTACCCCC	
dnmt3ab-qPCR-R	CGGACGATACCCACAGTGAT	
dnmt3bb.1-qPCR-F	AATGAGAACAGCCCCCTGAC	
dnmt3bb.1-qPCR-R	CGCTCCTGAAGACTTGTCCG	
dnmt3ba-qPCR-F	CGAAAGAGGACGACAACCGT	
dnmt3ba-qPCR-R	GTTCATGCCAGGCAGGTTTC	
dnmt3bb.2-qPCR-F	CTTTACCTGAACCGGGCACA	
dnmt3bb.2-qPCR-R	TATTCCCTGGAACGCACAGG	
baxa-qPCR-F	TGCATCAGATTCACGATGAGTT	
baxa-qPCR-R	ACGAGTCGGCATGCAAAGTA	
baxb-qPCR-F	TGGCAATAAAGCAGTGACGA	
baxb-qPCR-R	CCTCTCTTGTGGGACAAAGT	
caspase3a-qPCR-F	GGAAAACAATCAGCGGGCTC	
caspase3a-qPCR-R	CGTCAGTACCGTTTCGCTGA	
caspase3b-qPCR-F	ACTGTGGCTCAGATGAAAAAGC	
caspase3b-qPCR-R	GACCCTTGCAGTGGTTTCCT	
caspase8-qPCR-F	CCGCAACAGCAGTTCACATT	
caspase8-qPCR-R	TCAGGAAGAGGGGTGGGATT	
caspase9-qPCR-F	TTCCTAGTAAGCTATCGCCTGA	
caspase9-qPCR-R	CTATGTTGGAGCCCTTGCGA	
AMH-F5	ATGGCTCCGAGACCTTGACTG	Genetic sex identification
AMH-R3	CAGAAATGTAGACGCCCAGGTAT	
